# Orographic lift shapes flight routes of gulls in virtually flat landscapes

**DOI:** 10.1038/s41598-019-46017-x

**Published:** 2019-07-04

**Authors:** Elspeth Sage, Willem Bouten, Bart Hoekstra, Kees C. J. Camphuysen, Judy Shamoun-Baranes

**Affiliations:** 1 Theoretical and computational ecology, Institute for Biodiversity and Ecosystem Dynamics, Faculty of Science, University of Amsterdam, PO Box 94240, 1090 GE, Amsterdam, The Netherlands; 2 Department Coastal Systems, NIOZ Royal Institute for Sea Research and Utrecht University, P.O. Box 59, 1790 AB Den Burg, Texel The Netherlands

**Keywords:** Ecological modelling, Behavioural ecology

## Abstract

Interactions between landscape and atmosphere result in a dynamic flight habitat which birds may use opportunistically to save energy during flight. However, their ability to utilise these dynamic landscapes and its influence on shaping movement paths is not well understood. We investigate the degree to which gulls utilise fine scale orographic lift created by wind deflected upwards over landscape features in a virtually flat landscape. Using accelerometer measurements and GPS tracking, soaring flight is identified and analysed with respect to orographic lift, modelled using high-resolution digital elevation models and wind measurements. The relationship between orographic lift and flight routes suggests gulls have advanced knowledge of their aerial surroundings and the benefits to be gained from them, even regarding small features such as tree lines. We show that in a landscape constantly influenced by anthropogenic change, the structure of our landscape has an aerial impact on flight route connectivity and costs.

## Introduction

Landscape features play a vital role in facilitating the movement of species during many ecological processes, from foraging or migratory movements to reproduction and rest^1^. Anthropogenic landscape modifications can influence animal movements and thereby directly or indirectly affect species survival depending on the relationship an animal has with its environment^2^. Landscape modification may create barriers to movement between important ecological locations, or provide new landscape connectivity corridors, which will impact species distributions and ranges^3^.

Landscape connectivity can be defined as the ease with which individuals are able to move about the landscape, either directly due to habitat structure or through their behavioural response^4^. Studies of landscape connectivity have considered the impact of various landscape characteristics on terrestrial movement including vegetation^5^ and terrain variability^6^, whilst for aerial movement, wind conditions can create aerial corridors supporting long distance flights^7,8^. A measure of landscape connectivity through which such diverse environmental variables can all be expressed defines an energy landscape^9^, where landscape characteristics are measured in terms of their energetic impact. The energy landscape can help define the energetic cost of transport on a continuum^10^ either as a function of landscape features^11^ or directly through the impact landscape has on metabolic rate^12,13^.

Although landscape connectivity regarding terrestrial habitats is widely considered in current research^4^, the connectivity of atmospheric habitats^14^ has received less attention^15^, especially concerning fine scale interactions between landscape and atmosphere. The lower atmosphere is strongly influenced by surface processes^16^, giving rise to sources of uplift which contribute to an aerial energy landscape and which birds may exploit to save energy in flight^17^. Energy savings are gained through soaring flight, which is far less costly than flapping flight^18^, but relies on sources of uplift to maintain altitude. A notable source of uplift in this context is thermal lift, where birds will use rising air currents caused by surface heating to gain height^19^. For obligatory soaring birds, availability of thermal lift is known to shape migration corridors connecting distant breeding and non-breeding areas^20^.

Another source of atmospheric uplift utilised by soaring birds, orographic lift, results from upward deflections of moving air over physical obstacles^21,22^. The occurrence of such lift is highly dependent on landscape elevation and slope, so is predictable given consistent wind conditions. Reoccurrence of high orographic lift in the energy landscape can therefore be regularly exploited by soaring birds to save energy^23^, but so far has mainly been studied in mountainous areas in obligate soaring migrants at relatively coarse spatial scales^24–26^.

Whilst obligate soaring birds depend on aerial lift to remain airborne, for flight generalists who regularly use flapping and soaring flight, areas of reoccurring uplift present an opportunity to behave plastically according to internal motivation and available conditions^27–29^. Individuals may take advantage of uplift opportunistically when encountering it along their movement path. Furthermore, if individuals have knowledge of the distribution of uplift in the landscape, they could use this information to select flight routes. Birds are known to take cues from landscape features for various purposes, including navigation^30^, foraging^31^ and perceived threats^32^. Thus landscape features may influence movement decisions in different ways, depending also on an animal’s ability to map its environment^33^ and adapt to its changing dynamics. Artificial modification of landscapes by humans impacts the aerial environment, including the strength and distribution of orographic lift. If orographic lift is then utilised by birds for energy efficient soaring flight, landscape modification will impact flight energy budgets and could influence travel routes and foraging range. Thus understanding how birds utilise their aerial landscape will provide insight into the features shaping their daily movements and distributions, and the degree to which birds can adapt their flight behaviour to human induced landscape alteration.

This paper aims to determine the extent to which two flight generalist species utilise orographic lift across their terrestrial surroundings and the extent to which orographic lift shapes their flight behaviour and routes on a fine scale. Orographic lift in the landscape is examined on two temporal scales: an instantaneous scale, which describes the orographic lift a bird encounters at a given moment, and a time-independent seasonal scale, which describes the overall availability of orographic lift across the study period and identifies areas where orographic lift regularly occurs. Through these two measures we examine firstly whether birds select for orographic lift by comparing the orographic lift they encounter to orographic lift that would be encountered along alternative random flight routes, and estimate the relative energetic gain of the flight strategies used. We then examine whether the seasonal orographic landscape contributes to route formation and flight behaviour, and whether this gives insight into the ability birds have to map their landscape.

Two flight generalist gull species, the herring gull *Larus argentateus* and lesser black-backed gull *Larus fuscus*, are focused upon in this study. These highly opportunistic species have a diverse behavioural ecology and use primarily flapping flight, but are known to soar on thermal and orographic lift when possible^23,29,34^. Localised movements and flight behaviour are quantified through high-resolution GPS and accelerometer measurements collected over three consecutive breeding seasons across a landscape of lowlands surrounding the breeding colony, which are virtually flat and predominantly below sea level. Nevertheless, heterogeneous landscape features such as coastal dunes, raised roads and dikes are expected to provide enough orographic lift to support soaring flight. Behavioural decisions are examined in terms of flight behaviour, i.e. whether a bird chooses to soar or not, and the characteristics of the routes they take.

It is expected that gulls will select areas generating high orographic lift and that the probability of soaring will increase when enough orographic lift is available. Flight patterns are expected to demonstrate that gulls make route choices based on the seasonal orographic landscape. We discuss how human altered landscapes can influence the atmospheric landscape, in turn shaping the flight behaviour of birds and altering their energetic costs.

## Methods

### Ethics statement

All work conducted in the field to fit GPS-loggers to herring gulls and lesser black-backed gulls was carried out with approval from and in accordance with the Dutch ethics committee on animal experiments (DEC) of the Royal Netherlands Academy of Arts and Sciences (KNAW). Permission to work in the Kelderhuispolder gull colony was granted by the Stadsbosbeheer.

### Study area

The herring gulls and lesser black-backed gulls monitored for this study breed on the island of Texel, Netherlands, on an interface between the North Sea, Wadden sea, and mainland North-Holland. Whilst breeding these birds are central place foragers, making regular trips from their colony to terrestrial and marine locations to feed or rest^35–37^. Most terrestrial movement takes place south of the colony in North-Holland, a flat landscape predominantly at or below sea level, bordered by coastal dunes to the west and characterised by urban and agricultural developments.

### GPS data

Adult lesser black-backed gulls and herring gulls have been tracked using the UvA Bird Tracking System^38^ since 2008 and 2013 respectively as part of a long term study^29,35,37^. Data was selected from three consecutive breeding season (2015–2017), when large amounts of high-resolution data were collected, and included 30 individuals (21 herring gulls and 9 lesser black-backed gulls). GPS measurement intervals ranged between 3–600 seconds. All associated measurements, including orographic lift, were weighted by GPS measurement interval. Following every GPS measurement, tri-axial acceleration was measured at a frequency of 20 Hz for 1–3 seconds and used for behavioural classification. Behaviour was classified using a machine learning algorithm described in^29^. This algorithm was developed from accelerometer data of lesser black-backed gulls, but in this study was also applied to herring gulls to distinguish between three key activities: non-flight behaviour, flapping flight and soaring flight, as we expect herring gull movement to sufficiently reflect that of lesser black-backed gulls.

### Route and destination definition

Movement data was classified into trips, defined by an individual travelling further than 500 m from the colony (53.00 N, 4.72 E), staying away from the colony for at least one hour and travelling a cumulative distance of at least 4 km before returning. These constraints aimed to maximise trips associated with foraging movements. Trips where the proportion of time spent at sea exceeded 15% were removed, as orographic lift is not expected to be relevant for predominately marine based trips. Trips with less than 10 measurements or intervals between consecutive points of over 30 minutes were also removed. Within a trip gulls can utilise a wide range of foraging locations, stopping at various points in their journeys. Destinations within a trip were defined where the distance moved over a 15-minute interval either side of a GPS fix was less than 100 m. Destinations were used to retain objectives of trips when generating random routes for comparative analysis (see below for more detail). Data from 29 birds met these criteria, resulting in 231270 measurements for further analysis. Summary statistics were calculated per trip and are presented as mean ± standard deviation unless indicated as mean ± mean absolute deviation (MAD) where distributions were non-Gaussian. Trip travel duration and distance were defined as the sum of time intervals and cumulative point to point distances respectively, both excluding time spent at destinations. The percentage of detour per trip was quantified as cumulative point to point distance travelled minus shortest possible travel distance, divided by shortest possible travel distance.

### Generating random routes

To identify whether gulls select flight routes with orographic lift, real movement was compared to a movement model where birds were simulated to utilise the landscape randomly. A random route was generated for every real route, connecting the destinations along the route using a random trajectory model devised by^39^. This technique uses the time-stamps and average velocities of the real trips to generate maximum radii of travel between the closest known points, set initially to the trip origin and destination. The area of intersection enclosed within the maximum radial distances represents feasible movement locations, and within this area a random point is generated. This process is repeated between nearest points including newly generated points to produce a random route for each trip section between destinations. The resulting random route resembles the real route in that it fills the same time frame and visits the same destinations, but otherwise takes a random trajectory. Therefore destination goals are retained, whilst landscape utilisation is random. Speed was kept constant within each random route for ease of calculation, therefore where the time interval between GPS points was not constant in real routes, random routes had an equal time interval.

### Calculating orographic lift

For every position, of both real and random routes, an associated value of orographic lift was calculated, following^22^. Given a known wind speed and incident angle upon a feature of known elevation, slope and aspect, the upward deflected wind is calculated as the resultant orographic lift. A high-resolution LiDAR-based digital surface model^40^, at a 2.5 m scale resolution provided information on landscape height *z* and orientation, such that slope angle *θ* and aspect *β* could be calculated by the following equations:

1 $$\theta =\arctan {[{(\frac{dy}{dx})}^{2}+{(\frac{dz}{dy})}^{2}]}^{\frac{1}{2}}$$

2 $$\beta =\arctan [\frac{(\frac{dz}{dx})}{(\frac{dz}{dy})}]$$

Orographic lift *w*_*o*_was then calculated

3 $${w}_{o}=v(\sin (\theta )\cdot \,\cos (\alpha -\beta ))$$

Where *α* − *β* is the angle between the direction wind is blowing from and the direction the slope is pointing towards, and *ν* is wind speed.

Hourly wind data measured 2 m above surface was sourced from the Dutch Meteorological Institute KNMI^41^, using six local meteorological stations. For every GPS location wind data from the closest station and temporally nearest measurement was used, with the average distance between a GPS location and meteorological station being 6.8 km.

Within a 22.5 × 22.5 m grid around the GPS position, orographic lift was calculated per 2.5 m cell and the maximum value within the 22.5 × 22.5 m grid was used in further analysis. This was done to account for horizontal precision in the GPS measurement^38^ and to best represent the conditions available to a bird. In such a flat landscape taking mean orographic lift values would result in the elimination of most landscape effects.

### Data analysis

We hypothesise that individuals will select for orographic lift in their foraging flight movements and that this will be observed in the orographic lift they encounter as well as the amount of soaring they utilise. Therefore we expect to observe greater proportions of orographic lift in real routes compared to random routes, and a preference for soaring rather than flapping at low altitudes when orographic lift is strong enough to support soaring flight. Low altitude soaring is distinguished from other soaring as points where altitude above landscape elevation is ≤25 m. This value was chosen because orographic lift dissipates with height above the landscape; in the relatively flat dutch landscape we expect that soaring altitudes below 25 m represent soaring within the influence of orographic lift effects and that soaring flight at higher altitudes uses other sources of uplift, e.g. thermal convection. We compared the distribution of orographic lift in these low altitude soaring points with those of all real flight and of random flight, following^22^. Distributions of orographic lift are presented as relative probability distributions, scaled such that the area under each distribution is equal to 1. These distributions of orographic lift were compared using a Kolmogorov-Smirnov (KS) test, a non-parametric test which measures both scale and location of a distribution and does not require information on the underlying distribution shape. The statistical output of the KS test yields the maximal absolute difference between the distributions, *D*. For all flight points, a logistic regression was carried out to determine whether orographic lift experienced during flight had a significant influence on the probability of a bird using low altitude soaring.

In order to examine the spatial distribution of the seasonal orographic landscape, and investigate whether the seasonal landscape influences general movement decisions, a time-independent map of seasonal orographic lift was generated. Seasonal orographic lift was determined by calculating orographic lift averaged over time across the whole study area on a 22.5 × 22.5 m spatial grid using an hourly temporal resolution throughout the three breeding seasons studied. For each spatial grid cell the maximum orographic lift was taken per hour, as in previous analysis. These measurements were then averaged over time, and aggregated by mean onto cells of 125 × 125 m. The spatial distribution of terrestrial low altitude soaring was then determined by calculating the proportion of flight time spent on low altitude soaring per 125 × 125 m cell. This larger grid size was used to examine whether birds broadly seek areas where they anticipate favourable orographic lift conditions. To then test if gulls are more commonly moving through areas that on average have high orographic lift, seasonal orographic lift was spatially associated to all real and random flight points. The distribution of seasonal orographic lift was then compared between true flight and random flight using a KS test.

To test whether real routes contained more potential energy savings from orographic lift than random routes, we made an estimate of orographic lift strengths which represented conditions in which birds preferred to soar. This estimate was taken as the orographic lift values above which the probability of low altitude soaring exceeded 50%, as determined from the logistic regression model described above. In reality some soaring flight takes place below this value and some flapping flight above, but we expect this model to characterise preferred low altitude soaring opportunities. The total time spans of these soaring opportunities were calculated per trip, and their energetic costs compared in terms of basal metabolic costs (BMR) where soaring costs were assumed to be 2 x BMR^18^ and flapping costs 7 x BMR^42^ as in previous studies^29^. Cost differences in terms of BMR were determined per trip between real and random routes. For comparison to a landscape with no atmospheric effects and distance minimising flight, the transport cost in terms of BMR was calculated over the shortest possible travel distance per trip, assuming a bird only utilised flapping flight and travelled at minimum power speed, 10.4 ms^−1^ ^17^.

All data analysis was carried out in Matlab 2017a^43^, using the statistics and machine learning toolbox for statistical tests. Data visualisation was performed in Matlab 2017a^43^, R version 3.5.1^44^ and CloudCompare^45^.

## Results

**Figure 1:**
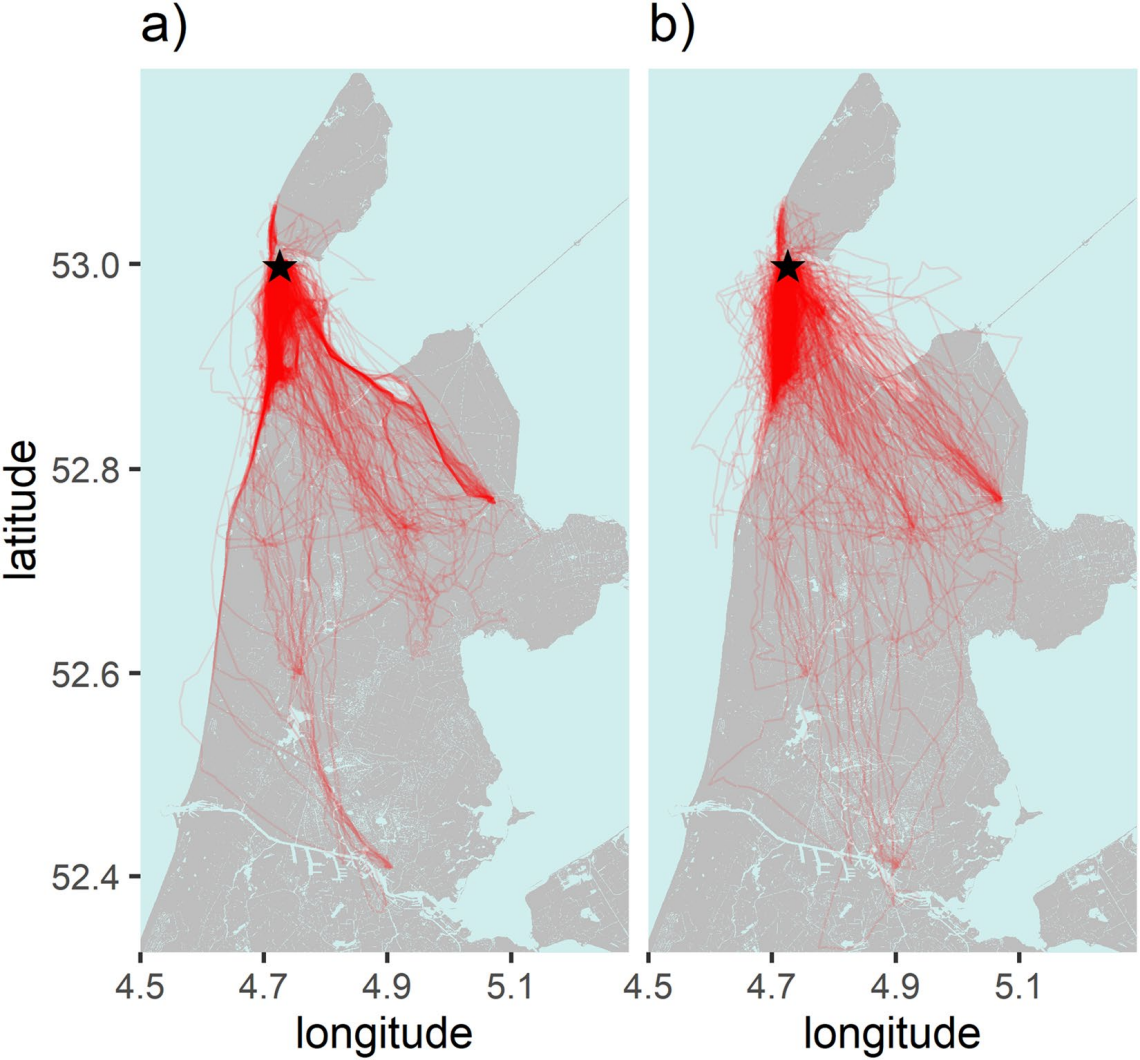
Spatial extent of (**a**) real routes taken and (**b**) random routes simulated for every real route. The centre of the breeding colony is indicated with a star. Land masses are represented in grey, water bodies in blue. Generated in R version 3.5.1^44^. Background maps are sourced from the Kadaster top10NL under CC-BY 4.0^56^.

Across all measurement points, 38.4% of time during flight was spent soaring and 63.8% of soaring flight occurred at low altitudes above the landscape. Altogether 816 trips were classified, ranging from 1 to 162 trips per individual. Mean travel time per trip (excluding time spent at destinations) was 2.1 ± 1.6 hours, with a mean travel distance of 29.7 ± 24.6 km and an average of 2.07 ± 1.36 destinations per trip. Birds took detours which resulted in real routes being on average 31.1 ± MAD 22.0% longer than the equivalent shortest path. A visual comparison of the real (Fig. 1a) and random routes (Fig. 1b) shows a far more orderly structure to the real trips, with regularly repeated routes taken, especially along coastlines and dikes.

### Instantaneous selection and utilisation of orographic lift

**Figure 2:**
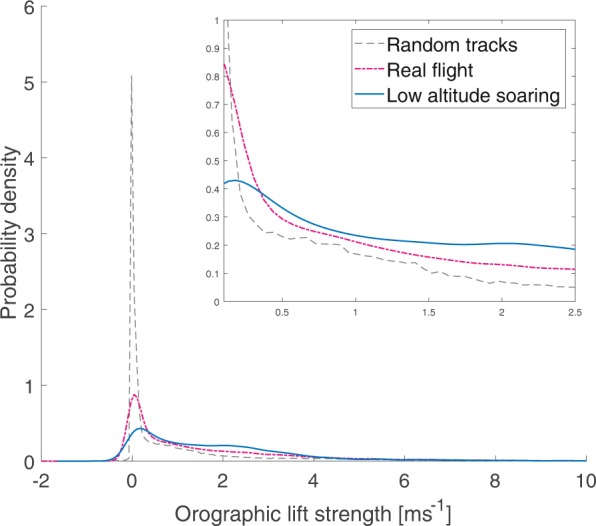
Relative probability density distributions of the orographic lift strength experienced at real flight locations (pink), the equivalent random route locations (grey dashed), and real locations of low altitude soaring (blue). Insert highlights data between orographic lift strengths of 0 and 2.5 ms^−1^.

We tested whether birds select for orographic lift by comparing orographic lift they encounter to orographic lift that would be encountered along alternative random flight routes, as measured on a 22.5 × 22.5 m scale. The resulting probability distributions of orographic lift in real flight, random flight, and real low altitude soaring differ significantly from each other (KS test between real flight and real low altitude flight: D = 0.2342, p < 0.0001, between real flight and random flight: D = 0.2318, p < 0.0001, Fig. 2). In random flight orographic lift is predominantly low in strength, and the probability of flight is highest where orographic lift is non-existent (−0.01 ms^−1^, Fig. 2), which is expected given the flat, relatively homogenous landscape profile of the study area. If flight routes were undertaken randomly then this would be the expected profile of orographic lift strength experienced by birds. However, in real flight orographic lift strength peaks at 0.04 ms^−1^ and the distribution of real flight exceeds that of random routes at higher orographic lift strengths. When only low altitude soaring flight is considered, a bimodal distribution is observed, peaking at 0.18 ms^−1^ and 2.00 ms^−1^, showing a non-random selection for locations with orographic lift strengths within range of the second peak. The relationships between these distributions therefore indicate that birds are making spatial decisions in order to move through areas where stronger orographic lift is available, and when they do so they switch from flapping to low altitude soaring. The logistic regression model showed orographic lift to be a highly significant predictor of the probability of low altitude soaring (Wald test, p < 0.0001). Based on the logistic regression model, the probability of soaring exceeded 50% above orographic lift strengths of 1.144 ms^−1^.

**Figure 3:**
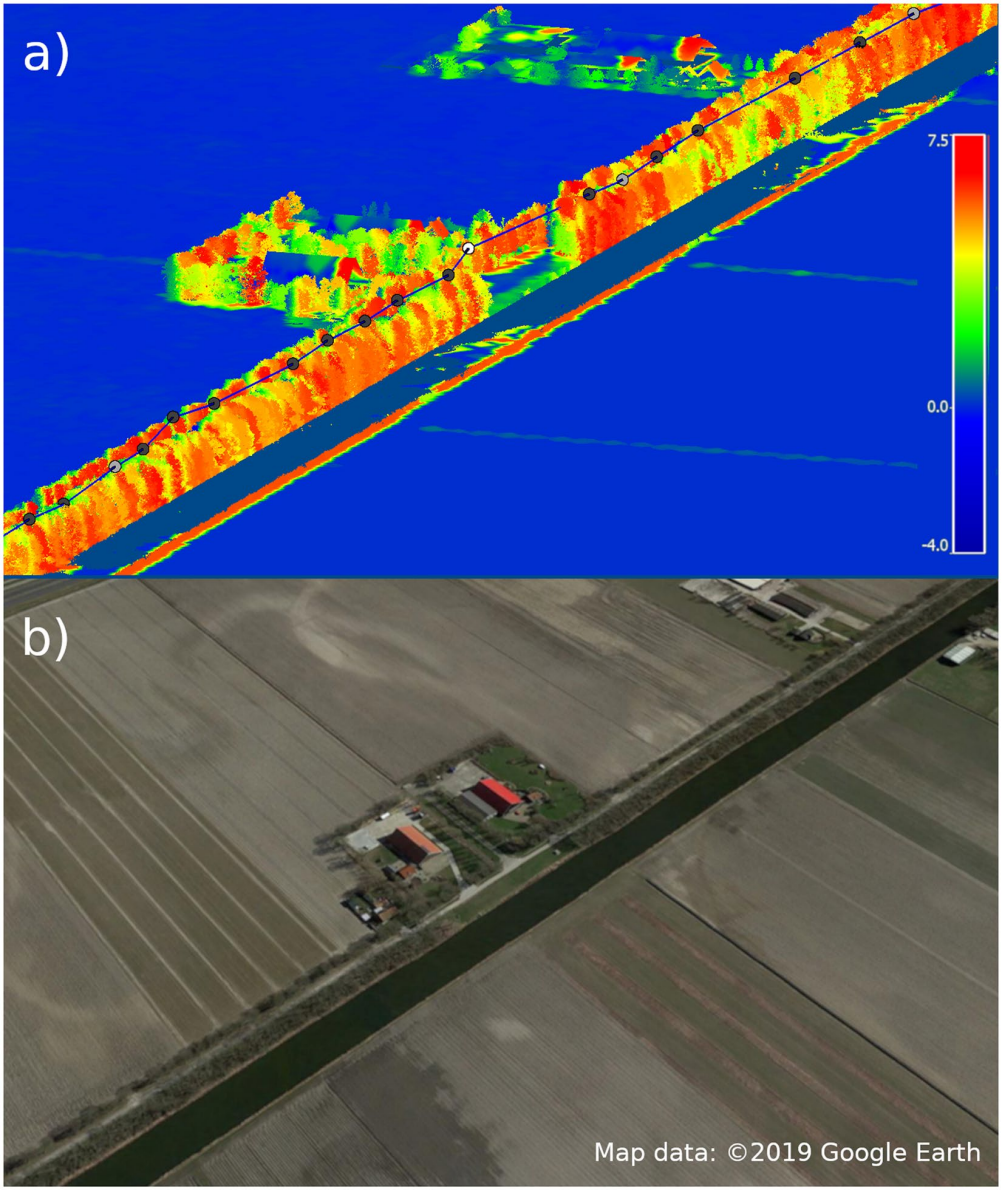
Fine scale response to orographic lift. (**a**) High-resolution GPS points taken every 3 seconds for individual 6202 undertaking low altitude soaring above a tree line, with soaring flight shown in black, flapping flight in white, and mixed flight in light grey. Orographic lift strength is calculated on a 2.5 m spatial resolution to the nearest hour is overlaid on a high-resolution LiDAR point cloud^40^. The relationship between flight position and behaviour and the orographic lift available is visualised, including behavioural reaction to a break in the tree line producing orographic lift. (**b**) Satellite image (Google Earth) of area, showing a canal and road lined by trees passing a farm in an agricultural area.

To provide insight into how gulls respond to orographic lift on a very fine scale, we illustrate in Fig. 3 an example of data points from a single trip measured at 3 second intervals. Orographic lift for this area was modelled to the nearest hour at a spatial resolution of 2.5 m, as dictated by the maximum DEM resolution, so that very fine changes in orographic lift could be observed. The gull soars at low altitude along a tree line, for which orographic lift is high in strength. When the tree line stops momentarily orographic lift likewise reduces, and the gull switches to flapping flight before returning to soaring flight over the tree line once again.

### Selection and utilisation of seasonal orographic lift

**Figure 4:**
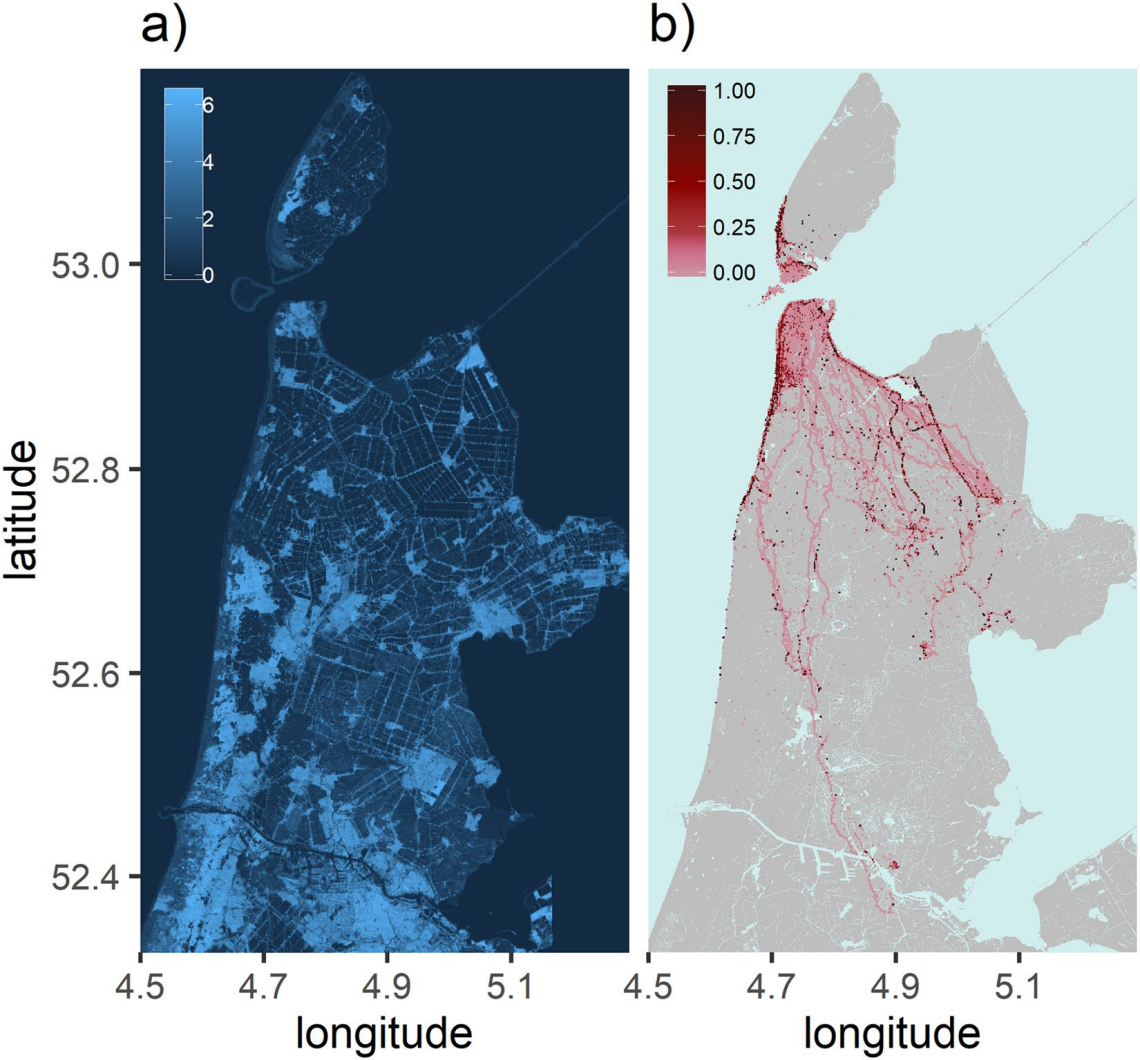
Spatial distribution of low altitude soaring and the orographic landscape. (**a**) Raster of seasonal orographic lift calculated per cell over the entire study period, based on hourly weather measurements. (**b**) Raster of proportion of time spent in low altitude soaring per cell, relative to the total time spent in flight per cell. Both raster maps were generated in R version 3.5.1^44^. Background maps are sourced from the Kadaster top10NL under CC-BY 4.0^56^.

**Figure 5:**
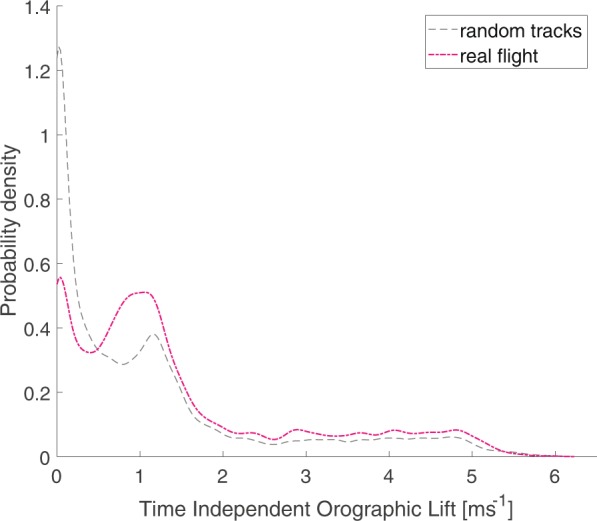
Relative probability distribution of the seasonal orographic lift experienced at real flight locations (pink) and at their random equivalents (grey).

A map of the seasonal orographic lift landscape is shown in Fig. 4a. Areas with high seasonal orographic lift reflect relatively large landscape features. In particular, large structures such as dunes, forests and urban areas appear as areas producing high orographic lift on average, which is partially a consequence of their capacity to generate orographic lift for multiple wind directions. Narrow, directional and continuous corridors of orographic lift are also observable; these features generally correspond to dikes, roads and tree lines. A map of the proportion of low altitude soaring per 125 m by 125 m grid cell across the study area is shown in Fig. 4b. High proportions of low altitude soaring were measured along areas of high flight convergence (see Fig. 1a) following directional and continuous landscape features that create corridors of orographic lift. In areas further from the colony with lower densities of GPS measurements of flight, the occurrence of low altitude soaring hot spots is more fragmented, likely due to the low number of points per cell. The relative probability distribution of seasonal orographic lift for true and random flight is shown in Fig. 5. Here it is seen that true flight differs significantly from random flight (KS test D = 0.1910, p < 0.0001) in terms of seasonal orographic lift. Specific peaks in the distributions are likely associated with areas which are repeatedly travelled over and which often produce a given orographic lift strength. At higher strengths of seasonal orographic lift, the likelihood of real flight occurring is greater than equivalent random points, indicating that birds are making non-random decisions regarding movement in terms of the seasonal orographic lift landscape.

Based on estimations of energy costs per trip for real and random routes, random routes were on average 22.75 ± 29.84% more costly. For a shortest route strategy in still air where gulls utilise only flapping flight at their minimum power speed, time spent travelling in trips was on average 65.4 ± 19.6% less, and were 3.78 ± 42.70% lower in energetic costs.

## Discussion

Our results show that herring gulls and lesser black-backed gulls are selecting flight routes so as to encounter above average orographic lift from the landscape, which even in a virtually flat landscape is sufficient to support soaring flight. Selection of orographic lift is illustrated firstly by the non- random distribution of orographic lift in true flight and particularly low altitude soaring flight shown in Fig. 2, indicating that gulls have knowledge of the orographic landscape and seek out locations of beneficial lift upon which to soar. The idea that gulls know this orographic landscape, also on a long temporal scale, is further supported in Fig. 5, where we observe that gulls travel more over areas which produce high orographic lift on average over time, as opposed to if they were moving between destinations randomly. By estimating the relative energy costs per trip when optimally soaring on orographic lift we see that preferential selection for orographic lift results in energy savings when compared to random routes. Gulls have long been observed soaring over landscape features, and whilst opportunistic gull flight over specific features generating lift has been studied^23,29^, this is the first time that the utilisation and selection of orographic lift on such a fine scale across a large spatial extent has been quantified.

In this study we see that at higher orographic lift strengths the probability of switching to low altitude soaring flight increases, whilst at intermediate orographic lift strengths the likelihood of a gull soaring versus employing alternate flight modes is roughly equal. When orographic lift strength is below a bird’s minimum sink rate, it can no longer gain altitude during soaring flight and would either lose altitude or have to switch to flapping or flap-gliding flight. Minimum sink rates modelled for herring gulls, (0.438 ms^−1^) and lesser black-backed gulls (0.469 ms^−1^)^17^ fall within the intermediate orographic lift strengths observed in this study, suggesting that the overlap of flight behaviours is related to whether a bird can maintain sufficient uplift above its sink rate. The actual uplift threshold at which a bird switches between flight modes will vary according to many additional factors, such as body morphology, internal drivers, and other environmental conditions such as horizontal winds or wind stability^23^. However, quick behavioural reactions to subtle changes in orographic lift strength demonstrate the sensory capability of gulls regarding air movements, and show that even relatively small landscape features such as tree lines are providing useful corridors of orographic lift.

These gulls do not take the most direct route between their colony and flight destinations, but follow repeated routes, converging upon paths which follow landscape structures. Our findings suggest that the motivation for taking these detours are at least in part explained by the availability of orographic lift which is used to enable soaring flight. When comparing the energetic cost of real flight routes and random routes, gulls save on average more than 20% of their energetic costs spent on flight. In the absence of wind, the shortest route would be faster and have slightly lower energetic costs than measured flights, and energy differences between real and shortest routes varied considerably. However the measures of energy savings calculated for shortest routes using flapping flight are highly simplistic and do not take into account effects of horizontal wind on flight behaviour and subsequent flight costs^46^. Other drivers of route formation may include motivation to gather information on resource availability. For central placed foragers who visit multiple destinations in a trip, feed on a wide array of resources and often behave opportunistically, exploratory behaviours in relation to resource distribution are expected to play a role in movement decisions^47^. Availability of lift may then make more distant sites more attractive or accessible and help facilitate the increased amounts of terrestrial foraging that are being observed in these species^36,48^. In addition, the role of landscape features in providing cues for animal navigation have been widely studied as a means by which routes are formed^49,50^. In order to obtain information about the landscape when forming routes, animals may use sensory or social information^51,52^, or a capacity to memorise their environment^53^. Based on their ability to select preferentially for locations producing orographic lift, this study indicates that gulls have a strong knowledge of their surroundings and the benefits to be gained from them.

In the context of landscape connectivity it is important to consider the role that orographic lift plays in creating corridors connecting habitats. This includes considering not just the strength, but also the spatial distribution of the orographic landscape. We see that many fragmented patches of high orographic lift are not necessarily utilised, as patches of orographic lift likely do not facilitate movement between locations in the same way that lines do if oriented along areas of interest. The most popular movement corridors, for flight in general and in particular for low altitude soaring, are dikes or tree lines. These features provide leading lines of orographic lift that birds will converge upon, as has been seen on larger scales when migrating raptors concentrate upon mountain ridges during migration^28,54^. Flight in the orographic landscape is therefore expected to be distributed according to the orientation of orographic lift corridors and in relation to the internal priorities of birds regarding their movement.

In highly anthropogenic landscapes which have been subject to much human alteration, even small changes to land cover alter the atmospheric resources available to volant species and by extension the energy landscape in which they move^9^. Landscape structure directly impacts the strength and distribution of orographic lift, which forms part of the energy landscape, and impacts landscape connectivity for soaring birds. Birds who use orographic lift to reduce their travel costs may gain energetic advantages in other aspects of their ecology, so changes to the orographic landscape may impact species range and distribution. A more comprehensive knowledge of the conditions in which orographic lift is used for movement will make it easier to predict the types of landscape characteristics which will attract the most convergence of movement, and allow us to predict flight paths with increasing accuracy across multiple species and scales^26^. In heavily developed landscapes, undergoing frequent human alteration, more detailed remote sensing techniques^55^ and more integrated studies of movement in relation to the landscape may improve our understanding of how specific landscape changes may alter the connectivity of movement paths. Assessments of the impact of landscape upon animal movement should then consider not just terrestrial movement and physical obstacles, but also the effect that landscape structure has on the atmosphere and the species that use it.

## Data Availability

Data available in the Dryad Digital Repository: 10.5061/dryad.pv229q5 (available upon publication).
